# Regulation and repurposing of nutrient sensing and autophagy in innate immunity

**DOI:** 10.1080/15548627.2020.1783119

**Published:** 2020-07-05

**Authors:** Julia Sanchez-Garrido, Avinash R. Shenoy

**Affiliations:** a Medical Research Council Centre for Molecular Bacteriology and Infection, Imperial College London, London, UK; b Satellite Group Leader, The Francis Crick Institute, London, UK

**Keywords:** AMPK, immunity, LC3-associated phagocytosis, microbial pathogenesis, MTOR, unconventional secretion

## Abstract

Nutrients not only act as building blocks but also as signaling molecules. Nutrient-availability promotes cell growth and proliferation and suppresses catabolic processes, such as macroautophagy/autophagy. These effects are mediated by checkpoint kinases such as MTOR (mechanistic target of rapamycin kinase), which is activated by amino acids and growth factors, and AMP-activated protein kinase (AMPK), which is activated by low levels of glucose or ATP. These kinases have wide-ranging activities that can be co-opted by immune cells upon exposure to danger signals, cytokines or pathogens. Here, we discuss recent insight into the regulation and repurposing of nutrient-sensing responses by the innate immune system during infection. Moreover, we examine how natural mutations and pathogen-mediated interventions can alter the balance between anabolic and autophagic pathways leading to a breakdown in tissue homeostasis and/or host defense.

**Abbreviations**: AKT1/PKB: AKT serine/threonine kinase 1; ATG: autophagy related; BECN1: beclin 1; CGAS: cyclic GMP-AMP synthase; EIF2AK4/GCN2: eukaryotic translation initiation factor 2 alpha kinase 4; ER: endoplasmic reticulum; FFAR: free fatty acid receptor; GABARAP: GABA type A receptor-associated protein; IFN: interferon; IL: interleukin; LAP: LC3-associated phagocytosis; MAP1LC3B/LC3B: microtubule associated protein 1 light chain 3 beta; MAP3K7/TAK1: mitogen-activated protein kinase kinase kinase 7; MAPK: mitogen-activated protein kinase; MTOR: mechanistic target of rapamycin kinase; NLR: NOD (nucleotide-binding oligomerization domain) and leucine-rich repeat containing proteins; PI3K, phosphoinositide 3-kinase; PRR: pattern-recognition receptor; PtdIns3K: phosphatidylinositol 3-kinase; RALB: RAS like proto-oncogene B; RHEB: Ras homolog, MTORC1 binding; RIPK1: receptor interacting serine/threonine kinase 1; RRAG: Ras related GTP binding; SQSTM1/p62: sequestosome 1; STING1/TMEM173: stimulator of interferon response cGAMP interactor 1; STK11/LKB1: serine/threonine kinase 11; TBK1: TANK binding kinase 1; TLR: toll like receptor; TNF: tumor necrosis factor; TRAF6: TNF receptor associated factor 6; TRIM: tripartite motif protein; ULK1: unc-51 like autophagy activating kinase 1; V-ATPase: vacuolar-type H^+^-proton-translocating ATPase.

## Introduction

Nutrient sensing is the fundamental process of detecting the availability of building-blocks ― including amino acids, sugars and lipids ― and governs whether cellular processes, such as growth and cell division, may proceed. The integration of nutrient sensing with growth factor and stress-response pathways is essential for cellular decision-making. In the presence of nutrients and growth factors, cells grow and proliferate. However, nutrients not only provide building blocks and energy to fuel cells, but also act as messengers that modulate signaling pathways. For instance, amino acids and glucose can influence global transcription, protein translation and organelle biogenesis by acting through regulatory kinases, such as MTOR (mechanistic target of rapamycin kinase) and the AMP-activated protein kinase (**AMPK**, see Glossary). MTOR and AMPK not only cross-regulate each other, but also orchestrate complex gene-expression programs and metabolic fluxes that shape cellular physiology. The absence of nutrients results in the shut-down of protein translation and stimulates **macroautophagy**/autophagy (see Glossary).

In the context of immunity, specialized immune cells must simultaneously evaluate nutrient availability, inflammatory cues and microbial signals. Recent reviews have focused on the metabolic regulation of various immune cell populations [1–5] and also on the cellular impact of MTOR, AMPK and autophagy in the immune system [5,6]. In this review, we provide a combined perspective of how signaling via pattern recognition receptor (PRRs) and cytokine receptors recruits the nutrient sensing and autophagy machineries to shape the immune response to microbes. We provide an overview of the signaling roles of nutrients such as amino acids, glucose and lipids and their roles in innate immune signaling. An appreciation of the crosstalk between anabolic processes and autophagy pathways helps better understand their re-wiring during infection and subversion by microbial pathogens.

## Nutrients as signaling molecules

Chemically diverse nutrients can influence immunity and inflammation. These processes are often manipulated by pathogens (as discussed below) and mutations in genes involved in the MTOR, AMPK and autophagy pathways are linked to immune deregulation and disease (Table 1). We first describe the mechanisms that detect amino acids, glucose and lipids and discuss the signaling outcomes of these processes in the next section.

**Table 1.** Nutrient sensing and/or autophagy-related genes with mutations linked to disease

Deregulation of:	Gene mutated	Disease-association	Reference(s)
Autophagy/Vesicle transport	*PLEKHM1*	Osteopetrosis	[181]
	*RAB7A*	Charcot-Marie-Tooth type 2B	[182]
Autophagy	*PLEKHM2*	Myocardial disorders	[183]
	*LAMP2*	Danon disease (cardiomyopathy)	[184]
	*PRKAG2*	Cardiac syndrome	[185,186]
	*ATG5*	Ataxia	[187]
	*ATG16L1*	IBD/Crohn disease, susceptibility to infection	[32,121,188]
	*CALCOCO2/NDP52*	Crohn disease	[189]
	*BECN1*	Cancer	[190]
	*PIK3R4/VPS15*	Cortical atrophy and epilepsy	[191]
Autophagy/inflammasome activation	*MEFV/TRIM20/Pyrin*	Familial Mediterranean fever	[83]
Autophagy (mitophagy)	*PINK1/PARK6*	Parkinson disease	[192–194]
	*PRKN/Parkin*	Parkinson disease, cancer, susceptibility to infection	[73,195,196]
	*LRRK2/PARK8*	Parkinson disease	[197,198]
	*OPTN1*	ALS, Primary open-angle glaucoma	[199–201]
Autophagy/MTOR signaling	*UBQLN2 and UBQLN4*	Familial ALS with or without FTD	[202,203]
	*SQSTM1*	FTD, ALS, Paget disease of the bone, DMRV, cancer, childhood-onset neurodegeneration	[57,204–207]
	*FLCN*	Birt-Hogg-Dubé syndrome.	[208]
Autophagy/MTOR signaling, innate immunity	*TBK1*	ALS, FTD, herpes simplex virus encephalitis, primary open-angle glaucoma	[209–211]
Lysosomal homeostasis, vesicle trafficking	*ATP6V1A*	Developmental encephalopathy, cutis laxa	[210,212]
MTOR signaling	*PIK3CA*	Cancer, PIK3CA-related overgrowth spectrum (PROS), Cowden-like syndrome	[213–215]
	*PIK3R2*	megalencephaly-polymicrogyria-polydactyly-hydrocephalus (MPPH)	[216]
	*PTEN*	PTEN hamartoma tumor syndrome (PHTS)	[217]
	*AKT1*	Proteus syndrome, Cowden-like syndrome	[215,218]
	*AKT2*	Hypoinsulinemic hypoglycemia	[219]
	*AKT3*	Hemimegalencephaly (HME), megalencephaly-polymicrogyria-polydactyly-hydrocephalus (MPPH)	[216,220]
	*DEPDC5*	Focal epilepsies; HCV-related hepatocellular carcinoma	[221–223]
	*NPRL2, NPRL3*	Focal epilepsies	[221]
	*TSC1-TSC2*	Tuberous sclerosis	[224]
	*MTOR*	Hemimegalencephaly (HME), focal cortical dysplasia (FCD), cancer	[220,225,226]
	*STK11/LKB1*	Peutz-Jeghers syndrome	[227]
	*CARD11*	Atopic dermatitis	[228]
Pro-survival signaling, inflammation	*CASP8*	IBD, autoimmune lymphoproliferative syndrome	[229,230]
	*RIPK1*	Immunodeficiency and IBD	[231]

**Abbreviations**: ALS: amyotrophic lateral sclerosis; DMRV: distal myopathy with rimmed vacuoles; FTD: frontotemporal dementia; IBD: inflammatory bowel disease.

### Amino acids

Leucine, arginine, methionine, glutamine and histidine, or their metabolites, signal through specific receptors that activate the MTOR complex 1 (**MTORC1** (see Glossary); also see Box 1). The following pathways activate MTORC1: 1) leucine levels directly detected by SESN2 (sestrin 2) and LARS1 (leucyl-tRNA synthetase 1); 2) acetyl-CoA produced from leucine metabolism [7]; 3) arginine detection by CASTOR1 (cytosolic arginine sensor for MTORC1 subunit 1) and the lysosomal amino acid transporter SLC38A9; 4) S-adenosyl methionine (SAM) sensing by BMT2/SAMTOR; 5) alpha-ketoglutarate produced through glutaminolysis of glutamine acting via prolyl hydroxylases (PHDs) and ARF1 (ADP ribosylation factor 1); 6) as yet unknown amino acids and homocysteine via the FLCN (folliculin)-FNIP1 complex. In contrast, a lack of amino acids results in the accumulation of uncharged tRNAs and ribosomal stalling, which activates EIF2AK4/GCN2 kinase activity [8].

### Glucose and glycolysis intermediates

The AMPK complex is activated by STK11/LKB1 in the presence of AMP, which signals reduced glucose availability and/or reduced ATP production through glycolysis and oxidative phosphorylation [9]. Glucose levels also modulate OGT (O-linked GlcNAc transferase), GAPDH (glyceraldehyde-3-phosphate dehydrogenase) and MLXIPL/ChREBP (MLX interacting protein like). OGT uses UDP-N-acetylglucosamine, the end product of the glucose-driven hexosamine biosynthesis pathway, to O-glycosylate transcription factors such as REL (a subunit of the NFKB complex) and MYC in macrophages, neutrophils and CD8^+^ T cells [10]. Notably, O-GlcNAc modification is essential for MAVS-dependent antiviral signaling [11] and the suppression of inflammatory necroptosis by RIPK3 [12] and thus links hexosamine biosynthesis to innate immune signaling. GAPDH, whose multiple functions depend on glycolytic flux, can bind to various mRNA, including *IL2* (interleukin 2) and *IFNG* (interferon gamma), and regulate their translation [13]. The glycolysis intermediate phosphoenolpyruvate regulates the activity of nuclear factors of activated T-cells (NFATs) in T cells by suppressing Ca^2+^ release from the ER [14]. Thus, in addition to regulating AMPK through ATP levels, glucose also regulates cellular responses through O-GlcNac modifications and altered glycolytic flux.

**Lipids**. Short-chain fatty acids (SCFAs) can be detected by G protein-coupled receptors (GPCRs) and histone deacetylases [15]; for example, propionate is detected by FFAR3/GPR41 (free fatty acid receptor 3) and FFAR2/GPR43, whereas butyrate, an anti-inflammatory molecule, is detected by HCAR2/GPR109A and histone deacetylases. Long-chain fatty acids (LCFAs), such as omega-3 fatty acids, are detected by FFAR1/GPR40 and FFAR4/GPR120, which results in ARRB2 (arrestin beta)-mediated sequestration of TAB2 and reduced pro-inflammatory cytokine production. Several fatty acids also regulate gene expression through peroxisome proliferator activated receptor (PPAR) transcription factors, among others [16].

Triglyceride-rich particles, including low-density lipoproteins (LDL), very-low-density lipoproteins (VLDL) and oxidized LDL (oxLDL), are detected by the phagocytic scavenger receptor CD36, which signals via non-receptor tyrosine kinases [17]. Sterols are sensed by SREBF2/SREBP2, SCAP, the insulin-induced gene complexes INSIG1 and INSIG2 and liver X receptors NR1H3/LXR-a and NR1H2/LXR-b [18,19]. AMPK and MTORC1 are key regulators of SREBF2 activation and integrate lipid metabolism with other pathways. Recent work has identified non-metabolic roles of SREBF2-SCAP, FFAR4 and FFAR1 in regulating the NLRP3 inflammasome [20–22]. For instance, cholesterol trafficking to the ER and SREBF2-SCAP are required for NLRP3 activation [20,22]. In contrast, FFAR4 and FFAR1 activation by omega-3 fatty acids suppresses NLRP3 by enhancing its binding to ARRB2 [21].

In the interest of space, we restrict our focus on amino acids and glucose/ATP and common themes in nutrient-dependent and -independent regulation of MTOR, AMPK and autophagy during infection and PRR signaling.

## Nutrient sensing and cellular responses

**Figure 1.**
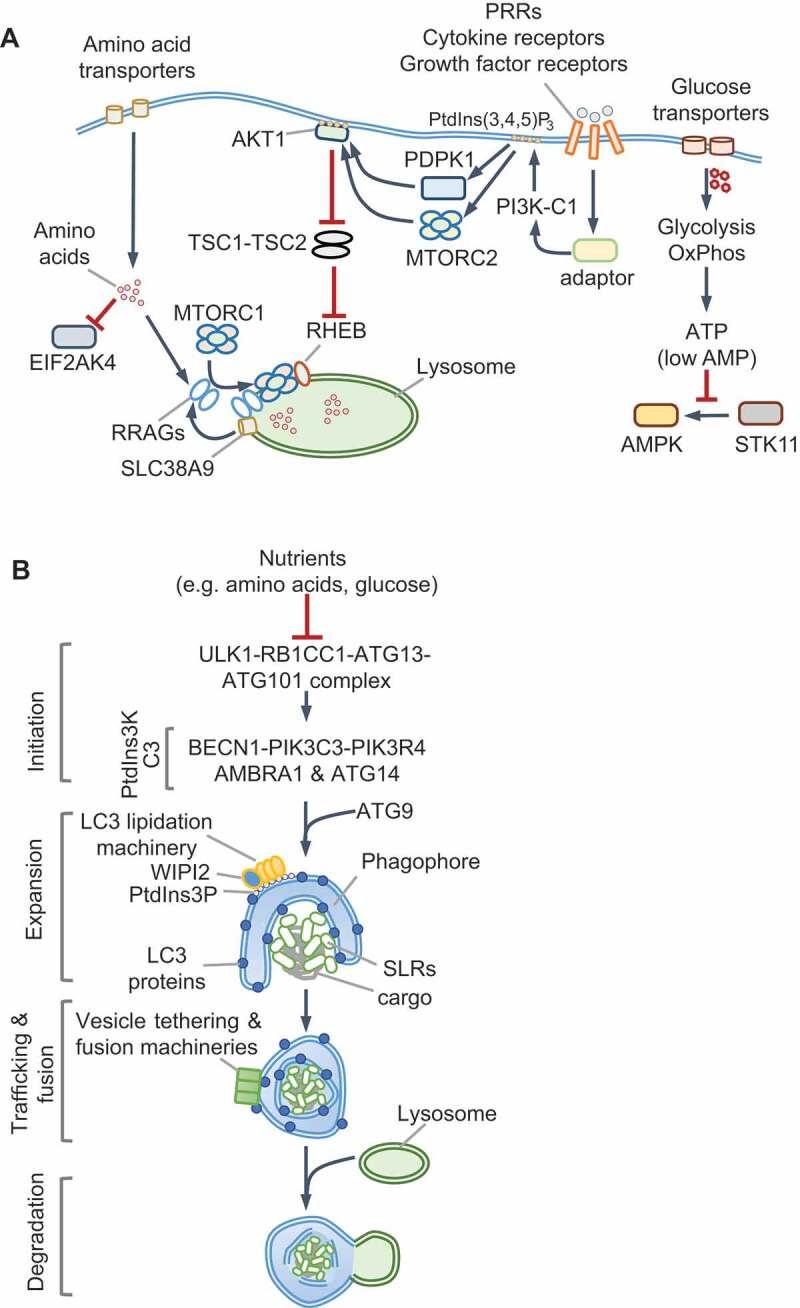
Nutrient sensing and autophagy. (A) Amino acids transported across the plasma membrane or from lysosomes are detected by various receptors (not shown; also see Box 1) that promote the lysosomal localization of MTORC1 and RRAG GTPases. Receptor signaling uses adaptors to activate class I PI3K (PI3K-C1), which produces PtdIns(3,4,5)P_3_ and activates AKT1 through PDPK1 and MTORC2. AKT1 phosphorylates TSC2 and inhibits its GAP activity toward RHEB GTPase. During amino acid starvation, uncharged tRNAs bind and activate EIF2AK4/GCN2. Glucose import stimulates enhanced ATP production through glycolysis and oxidative phosphorylation (OxPhos). In low-energy conditions, elevated cellular AMP binds AMPK, which results in its phosphorylation and activation by STK11/LKB1. (B) Nutrient starvation activates autophagy via the ULK1 complex by preventing its inhibition by MTORC1 and promoting its activation by AMPK. The steps involved in autophagy are labeled on the left. The BECN1-PIK3C3-PIK3R4 complex is a class III PtdIns3K (PtdIns3K-C3), which generates PtdIns3P on phagophores to recruit the Atg8 (LC3/GABARAP subfamilies) lipidation machinery, expand the membrane and engulf cargo. Ubiquitin-binding SQSTM1-like receptors (SLRs) and vesicle tethering and fusion machineries promote cargo-capture, vesicle transport and fusion with lysosomes. See Boxes 1 and 3. PI3K, phosphoinositide 3-kinase; PtdIns3P: phosphatidylinositol-3-phosphate; PtdIns(3,4,5)P_3_: phosphatidylinositol-3,4-5,-triphosphate; PtdIns3K: phosphatidylinositol-3ʹ-phosphate kinase

Nutrient sensing kinases MTOR and AMPK control the induction of key molecules that are co-opted by innate immune pathways. MTOR is a ubiquitously expressed, evolutionarily conserved eukaryotic serine/threonine kinase that forms two functionally distinct complexes, MTORC1 and **MTORC2**, which contain different proteins (see Glossary). MTORC1 responds to amino acids and growth factors and is activated through two analogous pathways that enlist GTPases, their GTPase activating proteins (GAPs) and guanine nucleotide exchange factors (Figure 1A and Box 1). Active Ras-related GTP-binding protein A (RRAGA), RRAGB and RHEB GTPases activate MTORC1 kinase activity on lysosomes. Normal lysosomal function is therefore essential for MTORC1 activation. Growth factor signaling triggers phosphatidylinositol-3,4,5-triphosphate (PtdIns[3,4,5]P_3_) production by class I phosphoinositide 3-kinase complex (PI3K-C1) and activation of MTORC2, which phosphorylates and activates AKT1 (Box 1). AKT1 inhibits TSC2 and thus indirectly activates MTORC1. Notably, amino acids, particularly leucine and glutamine, are essential for lysosomal MTORC1 localization to enable its activation by MTORC2-RHEB (for example, by INS [insulin]). The localization and activation of MTORC1 help integrate distinct signals for growth and proliferation. Interestingly, in response to EGF, but not insulin or amino acids, TBK1 (TANK binding kinase 1) phosphorylates MTOR on a newly identified site (Ser2159) and activates it independently of MTORC2–AKT1 [23], pointing to context-specific regulation of MTOR even by growth factors.

MTORC1 is the master regulator of anabolic processes, including protein, nucleotide and lipid synthesis, and an inhibitor of autophagy and lysosome biogenesis. These processes contribute to MTORC1-driven changes in cell volume, growth and proliferation and rely on amino acids as signaling molecules as well as carbon and nitrogen sources. MTORC1 controls the cellular proteome by enabling 7-methyl-G(5ʹ)ppp (also called 5ʹ-CAP)-dependent translation via EIF4E (eukaryotic initiation factor 4E; Box 2). EIF2AK4 activation by amino acid starvation leads to the phosphorylation and inhibition of EIF2S1 (eukaryotic initiation factor 2 subunit 1), a broad shut-down of protein translation and initiation of the integrated stress response. However, translation of the transcription factor ATF4 is not impeded, which enables the selective expression of genes involved in amino acid biosynthesis [24,25]. EIF2AK4 and MTORC1 thus coordinate the proteome and transcriptome under contrasting conditions.

AMPK orchestrates increased glycolysis and oxidative phosphorylation through glucose transporters SLC2A1/GLUT1 and SLC2A4/GLUT4, and transcriptional changes through PPARGC1A (peroxisome proliferator-activated receptor gamma, coactivator 1 alpha), HDAC5 (histone deacetylase 5) and SIRT1 (sirtuin 1) for increased mitochondrial biogenesis and beta-oxidation of fatty acids, all of which together increase cellular energy levels. AMPK suppresses anabolic processes, including cholesterol and lipid biogenesis, by inhibiting SREBF1, ACACA/acetyl-coenzyme A carboxylase (acetyl-CoA carboxylase 1 alpha) and ACACB among others. A key protein activated by AMPK is the serine/threonine kinase ULK1 (unc-51 like kinase 1), a principal component of the autophagy initiating **ULK1 complex** (see Glossary), which can also be inhibited by phosphorylation by MTORC1. The crosstalk between AMPK and MTORC1 is therefore important for homeostasis. Below, we discuss the regulation of autophagy initiation by AMPK and MTORC1, which is followed by sections on how these pathways are repurposed by innate immunity.

## Autophagy and lysosomal degradation

For details on the core autophagy pathway in starved cells, we refer readers to recent reviews [26–28] (also see Box 3). Bulk autophagy, aggrephagy (removal of protein aggregates), mitophagy (removal of mitochondria), pexophagy (removal of peroxisomes) and xenophagy (removal of intracellular microbes) involve the sequential activation of protein complexes for the formation of phagophores containing phosphatidylinositol-3-phosphate (PtdIns3P), which recruits machineries for membrane expansion, cargo capture and engulfment into double-membrane autophagosomes and their fusion with lysosomes (Box 3, Figure 1B). During starvation, the ULK1-ATG101-ATG13-RB1CC1/FIP200 kinase-containing complex initiates canonical autophagy by recruiting and activating the BECN1 (beclin 1)-PIK3C3/VPS34 (phosphatidylinositol 3-kinase catalytic subunit type 3)-PIK3R4/VPS15-ATG14 complex, which is a class III phosphatidylinositol 3-kinase complex (**PtdIns3K-C3**, see Glossary) that generates PtdIns3P. Lipidation of proteins of the **Atg8 family** (LC3 and GABARAP subfamilies, see Glossary), through conjugation to phosphatidylethanolamine by the Atg8-lipidation machinery, is required for the degradation of the inner autophagosomal membrane and the fusion of autophagosomes with lysosomes, but is not essential for autophagosome formation and autophagy [29,30] (Box 3). In mammals, ATG proteins and the Atg8-family proteins are additionally involved in other cellular pathways aside from canonical autophagy. For example, ATG16L1 plays an important role in Ca^2+^-mediated plasma membrane repair, independently of LC3, and can also regulate LC3 lipidation on single membranes; a process that specifically requires its WD40 domain [31,32]. Therefore, not all LC3-positive membranes are necessarily involved in canonical autophagy. As discussed in the following sections, in innate immune cells the recruitment of the LC3-lipidation machinery may involve distinct initiation steps and/or PtdIns3K-C3 complexes, and their differential regulation.

AMPK and MTORC1 receive nutrient and growth factor cues and respectively activate or inhibit the ULK1 complex. ULK1 phosphorylates and activates other proteins within the complex as well as BECN1, PIK3C3 and AMBRA1 [33]. In addition, ULK1 inhibits MTORC1 by phosphorylating RPTOR/Raptor, which illustrates feedback regulation. Autophagy-mediated breakdown and release of amino acids, such as glutamine [34], from lysosomes reactivates MTORC1 and turns off autophagy by inhibiting ULK1. In addition to phosphorylation, the appropriate localization of ULK1 and BECN1 complexes is key for selective autophagy. Cargo ubiquitination by E3 ubiquitin-ligases and their detection by ubiquitin-binding SQSTM1/p62-like receptors (**SLRs**) [35] helps recruit the autophagy machinery to selective cargo. Altogether, the core autophagy cascade follows once set in motion by the ULK1 and PtdIns3K-C3 complexes (Figure 1B), which also function as major regulatory hubs in response to infection [26,27]. Below, we discuss recent work on how the differential spatiotemporal regulation of these molecules redirects autophagy and nutrient sensing in response to PRR signaling and cytokines. Importantly, studies on innate immunity highlight mechanisms of dual activation of AMPK-MTORC1 and autophagy for host-defense and homeostasis.

## Repurposing of nutrient sensing pathways

Various cell types encounter microbes and provide the first line of defense. These include phagocytic cells such as macrophages, dendritic cells (DCs) and neutrophils, and epithelial cells lining the gut, lung and skin. These cells can detect infection or microbial products and inform other cells by producing inflammatory cytokines and launching cell-intrinsic growth-restriction mechanisms. Below, we discuss the regulation of nutrient sensing and autophagy by primarily focusing our attention on signal transduction mechanisms and key regulatory hubs within these pathways during innate immune signaling.

### Nutrients, STK11 and AMPK

Although STK11/LKB1 phosphorylates and activates AMP-bound AMPK [36], STK11 has nutrient and AMPK-independent actions in macrophages and DCs. For example, STK11 can bind to IKBKB/IKKB (inhibitor of nuclear factor kappa B kinase subunit beta) and inhibit NFKB signaling in LPS-treated macrophages [37]; however, prolonged signaling results in N-nitrosylation and degradation of STK11 [38]. The role of AMPK in these settings is currently unknown.

**Figure 2.**
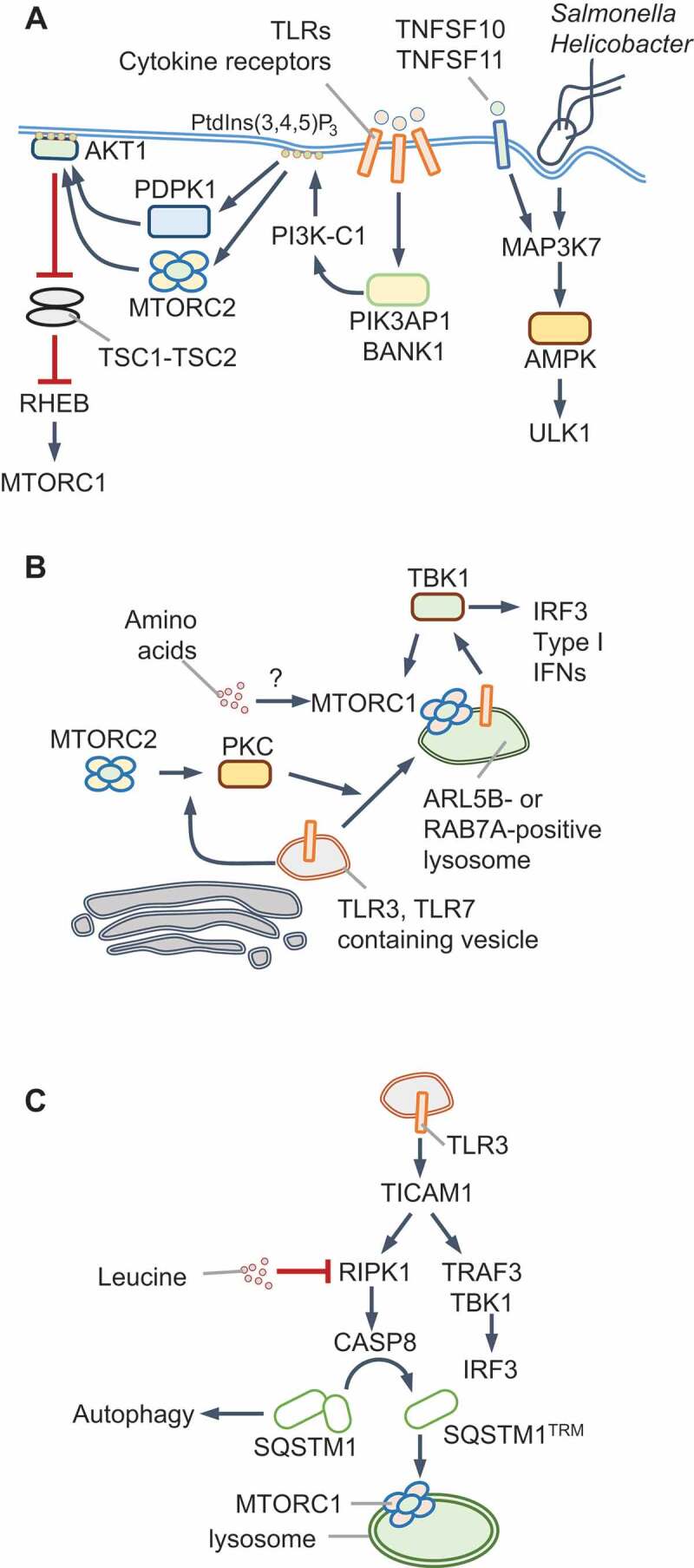
MTORC1 and AMPK regulation during infection and innate immune signaling. (A) TLR activation results in the recruitment of the adaptors PIK3AP1/BCAP or BANK1 that activate PI3K-C1 and PtdIns(3,4,5)P_3_ production. Infection by *Salmonella* or *Helicobacter* or exposure to TNFSF10/TRAIL, TNFSF11/RANKL and TLR9 stimulate MAP3K7/TAK1-dependent phosphorylation and activation of AMPK, which stimulates ULK1-dependent autophagy. (B) Activation of endosomal TLRs such as TLR3 or TLR7 results in MTORC1-dependent activation of TBK1 and type I IFN production. During TLR3 signaling, PKC activation by MTORC2 promotes trafficking to cell periphery to lysosomes that are positive for RAB7A. TLR7 activation results in its trafficking to ARL5B/ARL8-positive lysosomes for TBK1 activation. TBK1 may also contribute to MTORC1 activation. Amino acids may promote lysosomal localization of MTORC1; however, this remains to be tested. (C) TLR3 signaling through its adaptor TICAM1/TRIF triggers the cleavage of SQSTM1/p62 via RIPK1 and CASP8 at Asp329. The trimmed protein, SQSTM1/p62^TRM^, is required for MTORC1 activation in response to amino acids and leucine. Full-length SQSTM1 selectively participates in autophagy maturation, whereas SQSTM1/p62^TRM^ promotes MTORC1 and suppresses autophagy

AMPK can also be activated independently of STK11 and AMP by calcium flux-activated CAMKK2 and MAP3K7/TAK1 (mitogen-activated protein kinase kinase kinase 7) in response to TNFSF10/TRAIL (TNF superfamily member 10) and TNFSF11/RANKL (Figure 2A) [39–42]. In agreement with MAP3K7-driven AMPK regulation, MAP3K7-deficiency in hepatocytes increases MTORC1 activity and reduces autophagy, which can be restored by ectopic AMPK activation [43]. Infection by *Salmonella enterica* Typhimurium or *Helicobacter pylori* triggers MAP3K7-dependent AMPK activation, which promotes autophagy and host defense [44,45]; however, the precise mechanisms of AMPK activation remain unclear (Figure 2A). Together, these pathways exemplify nutrient-independent deployment of AMPK in shaping the cellular environment through ULK1 activation and MTORC1 inhibition.

### Regulation of PI3K-C1, MTORC1 and MTORC2

Fine-tuning of PI3K-C1, MTORC1 and MTORC2 helps shape immune responses by PRRs and IFNs, and we highlight key themes below.

#### Class I PI3K complex (PI3K-C1)

Growth factor receptors use the phosphotyrosine-binding Src homology 2 (SH2)-domain and SH3 domain-containing adaptor GRB2 (growth factor receptor-bound protein 2) or the SH2-containing PIK3R1/p85 subunit of PI3K-C1 to recruit and activate the lipid kinase complex. The resulting increase in plasma membrane PtdIns(3,4,5)P_3_ activates MTORC2 by binding to MAPKAP1/mSIN1, a specific subunit of MTORC2 [46]. Downstream activation of AKT1 inhibits **TSC1-TSC2** (see Glossary) and thus activates MTORC1, thereby linking receptor-stimulation to MTORC1 activation (Box 1). TLR and IFN signaling mirror growth factor receptor activation during nutrient-independent activation of PI3K and MTORC2. For example, tyrosine phosphorylation of Janus kinases (JAKs) activates PI3K-C1 directly and through GRB2. The TLR and IL1R family receptors, which do not undergo tyrosine phosphorylation, instead use the TIR domain-containing adaptors PIK3AP1/BCAP (phosphoinositide-3-kinase adaptor protein 1) and BANK1, which undergo tyrosine phosphorylation, to mediate PI3K-C1 regulation [47–51] (Figure 2A). For example, in plasmacytoid DCs (pDCs), SRC and LYN tyrosine kinases are constitutively active and can phosphorylate PIK3AP1 for PI3K-C1-MTORC1 activation [52]. We speculate that TLR/IFN signaling promotes MTORC2-driven responses and that nutrient signals acting on MTORC1 enable its lysosomal localization and activation.

#### MTORC1 and MTORC2

The metabolic consequences of MTORC1 or AMPK activation in myeloid cells have been discussed before [53]. Therefore, here we focus on signal transduction mechanisms and subcellular cues that link MTOR kinase complexes to PRRs and IFNs. The endosomal TLRs, TLR3 and TLR7, activate MTORC1 on the lysosome, which is essential for type I IFN production [54–56]. TLR3 traffics to RAB7A-positive lysosomes and TLR7 to ARL5B/ARL8 (ADP ribosylation factor like GTPase 5B)- and PLEKHM1 (pleckstrin homology and RUN domain containing M1)-positive lysosomes for MTORC1 activation (Figure 2B). Although not formally tested, it is plausible that nutrients license the lysosomal transfer of MTORC1 through RRAG GTPases. These studies also revealed that MTORC1 is dispensable for NFKB-dependent CCL5 and IL12p40 production, suggesting that nutrient-input is a dominant checkpoint for IFN responses, but not for pro-inflammatory cytokine and chemokine production during TLR3 signaling.

Work from our laboratory showed that TLR3 triggers RIPK1 and CASP8 (caspase 8)-dependent cleavage of a portion of cellular SQSTM1/p62 into a new MTORC1-regulatory molecule called SQSTM1/p62^TRM^ (amino acids 1–329), whose production correlates with the sustained activation of MTORC1 and RPS6KB1/p70S6K1 phosphorylation [57] (Figure 2C). Leucine starvation similarly generates SQSTM1/p62^TRM^ for MTORC1 activation when leucine becomes available. MTORC1 activation by TLR3 and leucine requires the scaffolding role of RIPK1 and catalytic activity of CASP8 [57]. Interestingly, a complex containing the paracaspase MALT1 (mucosa-associated lymphoid tissue lymphoma translocation protein 1) and CARD11/CARMA1 (caspase activation and recruitment domain containing protein 1) promotes MTORC1 activation independently of BCL10, which is normally required for the formation of the tripartite CBM signaling complex [58,59]. The requirement for the catalytic activity of the paracaspase MALT1 for MTORC1 activation mirrors the role of CASP8 in MTORC1 signaling [57]. Furthermore, the MTORC1-specific subunit RPTOR contains a caspase-like domain [60]. These surprisingly deep evolutionarily links between MTORC1 and caspases deserve further examination to determine their functional interplay in homeostasis.

Cytosolic DNA is sensed by CGAS/cGAS (cyclic GMP-AMP synthase), which activates STING1 (stimulator of interferon response cGAMP interactor 1) and TBK1 for type I IFN production. Notably, TBK1 can phosphorylate MTOR on Ser2159 for enhanced type I IFN production [23]. The role of nutrients in targeting MTORC1 to lysosomes during cytosolic DNA sensing has not been studied; however, abnormal lysosomes disrupt MTORC1 signaling and IFN production. For instance, loss of the lysosomal nuclease TREX1 results in increased accumulation of undigested DNA, lysosomal dysfunction and inflammatory disease driven by type I IFNs as well as hypermetabolic disease due to MTORC1 inactivation [61,62]. Fine-tuning of lysosomal MTORC1 activity, normally through nutrient input, thus maintains homeostasis during cytosolic DNA sensing.

Type I and type II IFNs co-opt both MTORC1 and MTORC2 for transcriptional changes. Silencing or genetic loss of MTORC2-specific proteins *Rictor* or *Mlst8* inhibits interferon-stimulated gene (ISG) expression [63,64]. Interestingly, several ISGs (e.g., IDO1 [indoleamine 2,3-dioxidase] depletes amino acids) promote a state of regulated starvation that reduces long-term MTORC1 activity. Indeed, polysome analyses of IFNG-stimulated cells suggest inhibition of MTORC1 based on global suppression of 5ʹ-terminal oligopyrimidine (TOP) motif-containing mRNA, which includes nutrient transporters, leucyl-tRNA and the transcriptional repressor HES1 (hes family bHLH transcription factor 1). At the same time, IFNG selectively enhances the translation of inflammatory cytokines and chemokines. These findings indicate fine-tuning of the proteomic landscape through nutrient-signals and MTORC1 during IFNG responses [65]. Reduced MTORC1 activity correlates with higher autophagy in IFNG-stimulated cells [66], which may promote xenophagy-mediated cell-intrinsic immunity. Such responses could be further facilitated by IFNG-induced GBPs (guanylate-binding proteins) [67] and tripartite motif-containing proteins (TRIMs) [68]. Thus, in addition to upregulating a large number of new proteins, IFNG also fine-tunes nutritional homeostasis and autophagy through the collective actions of these proteins. In summary, the activation of innate immune membrane-bound or cytosolic sensors can co-opt AMPK and/or MTORC1 pathways to drive an optimal cellular response.

## Repurposing of autophagy-related machinery

In this section, we discuss autophagy-related processes, such as LC3-associated phagocytosis (LAP) and unconventional protein secretion, that are independent of nutrient control. Autophagy-mediated regulation of inflammation, cell death and immune cell function are reviewed elsewhere [6,69]. Here, we focus on recent work on the differential localization and regulation of xenophagy initiation via ULK1-BECN1-dependent and -independent mechanisms. As discussed below, MTORC1-independent autophagy may simultaneously enable nutrient-independent repurposing of autophagy and MTORC1-driven transcriptional programs.

### Regulation of ULK1 and BECN1-PtdIns3K-C3 complexes

**Figure 3.**
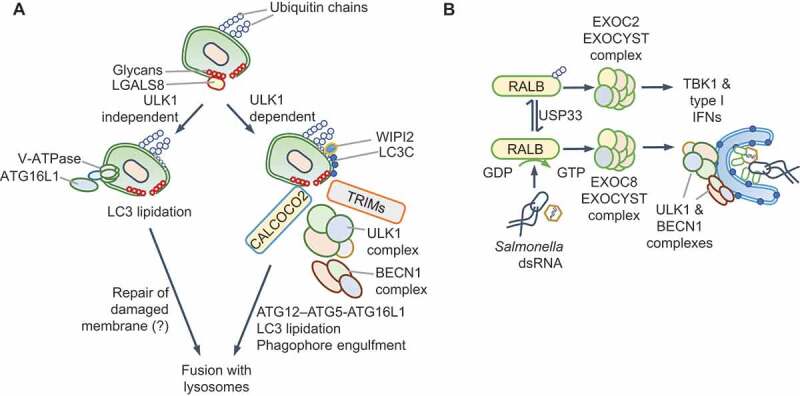
Redirection of ULK1 and BECN1-PtdIns3K-C3 complexes. (A) Damage of pathogen-containing vacuoles exposes luminal glycans that recruit galectins. Galectins, such as LGALS8 (galectin 8) and LGALS3 (galectin 3), can recruit the ULK1 complex through the SQSTM1-like protein (SLR) CALCOCO2/NDP52. TRIMs can detect microbial components or endosome damage through galectins and recruit the ULK1 complex. The subsequent recruitment of BECN1-PtdIns3K-C3 complexes promotes ATG12–ATG5-ATG16L1-driven LC3 lipidation. Ubiquitination of pathogens or associated membranes with polyubiquitin chains of different topologies further enhances the recruitment of the autophagy machinery through SLRs. In contrast, the V-ATPase complex can directly recruit ATG16L1 for MAP1LC3B (LC3B) deposition on *Salmonella*. Both pathways result in vacuole maturation, fusion with lysosomes and restriction of bacterial replication. (B) During infection by *Salmonella* or RNA virus or exposure to cytosolic dsRNA, the RALB GTPase differentially promotes autophagy and innate immune signaling. Active RALB promotes xenophagy through the EXOC8 subcomplex of the EXOCYST, which activates ULK1 and BECN1. RALB can be ubiquitinated at Lys47, which sterically hinders its interaction with EXOC8 and enhances binding to the EXOC2 subcomplex of the EXOCYST. This complex promotes the activation of TBK1 and type I IFNs during viral infection. USP33 can remove ubiquitin chains and alter the balance between the two pathways

Recent work has revealed that localization of autophagy-initiating complexes to intracellular pathogens promotes xenophagy and restricts bacterial replication. Damage to pathogen-containing vacuoles or lysosomes triggers autophagy through conventional or ULK1-independent routes. Galectins are a family of nine beta-galactoside sugar-binding proteins in the human, which detect luminal glycans exposed to the cytosol as a consequence of damage to endogenous vesicles [70]. In response to damage to endosomes or lysosomes, galectins stimulate autophagy through MTOR and AMPK; for example, LGALS8 (galectin 8) inhibits MTOR, and LGALS9 (galectin 9) activates AMPK in response to lysosomal damage [71]. However, early during microbial infection, nutrients are not limiting and MTORC1 inhibition is not a pre-requisite for xenophagy initiation. The ULK1 and BECN1-PtdIns3K-C3 complexes can traffic to intracellular bacteria upon pathogen-detection by E3 ubiquitin ligases [72–77], galectins [78,79] or proteins of the tripartite motif family (TRIM proteins) [68,80–83] (Figure 3A). The PtdIns3K activity of the PtdIns3K-C3 complex can then recruit PtdIns3P-binding proteins such as WIPI2 (WD repeat domain, phosphoinositide interacting 2) and the LC3 lipidation machinery for autophagy maturation. An exciting new model of xenophagy involves the direct recruitment of ATG16L1 to *Salmonella-*containing vacuole through its interaction with the vacuolar V-ATPase [84]. MAP1LC3B (LC3B) deposition on *Salmonella* can therefore also proceed independently of ULK1. An unanswered question is whether, in this scenario, LC3B is lipidated on single or double membranes, the latter being common in canonical xenophagy [84] (Figure 3A). Similarly, ULK1- and MTORC1-independent mitophagy or pexophagy can be engineered by the synthetic localization of CALCOCO2/NDP52, an SLR, to these organelles [85]. These findings suggest that the wider involvement of ULK1-independent LC3 lipidation, which is therefore independent of MTORC1, in xenophagy requires further attention. These studies have shown that targeting the autophagy initiating machinery to microbial vacuoles is critical for xenophagy.

Recent work also highlights the importance of regulating the activities of the ULK1 and BECN1-PtdIns3K-C3 complexes to fine-tune xenophagy. Although several TRIM proteins detect microbial signals and recruit ULK1-BECN1 and the LC3 lipidation machinery (Figure 3A), others such as TRIM23 promote autophagy maturation by promoting SLR phosphorylation [86]. The RALB GTPase is another example of a regulatory switch that controls ULK1 activity. RALB associates either with EXOC2/SEC5 and EXOC8/EXO84, which are two components of the **EXOCYST** (see Glossary) complex [87]. RALB-EXOC8 interaction promotes ULK1 and PtdIns3K-C3 activity, for example, during *Salmonella* and Sendai virus infection [88] (Figure 3B). In contrast, RALB-EXOC2 interaction inhibits ULK1, but promotes EXOC2-TBK1-driven antiviral IFN production [89] (Figure 3B). RALB, which also controls starvation-induced autophagy, is thus co-opted during infection for xenophagy or IFN production.

A third protein that regulates autophagy initiation through BECN1 is TRAF6, a ubiquitin E3 ligase. TRAF6 mediates K63-linked ubiquitination of BECN1 and promotes autophagy [90–92] by suppressing BECN1 binding to its inhibitor BCL2 [90,93]. Surprisingly, TRAF6 also modifies MTORC1 with K63-linked ubiquitination in response to amino acids, promoting its activation. TRAF6 may therefore integrate immune signaling and metabolic cues to stimulate autophagy or MTORC1 in a context-dependent manner [94].

Taken together, localization and regulation of ULK1 and BECN1-PtdIns3K-C3 complexes through SLRs, TRIMs, RALB and TRAF6, and possibly other yet to be discovered proteins, are key to the dual control of autophagy and MTORC1. As discussed next, cytosolic DNA sensing has emerged as a pathway that bypasses ULK1 and BECN1 for autophagy initiation independently of MTORC1 as a gatekeeper.

### Multiple roles of STING1 and TBK1 in autophagy

**Figure 4.**
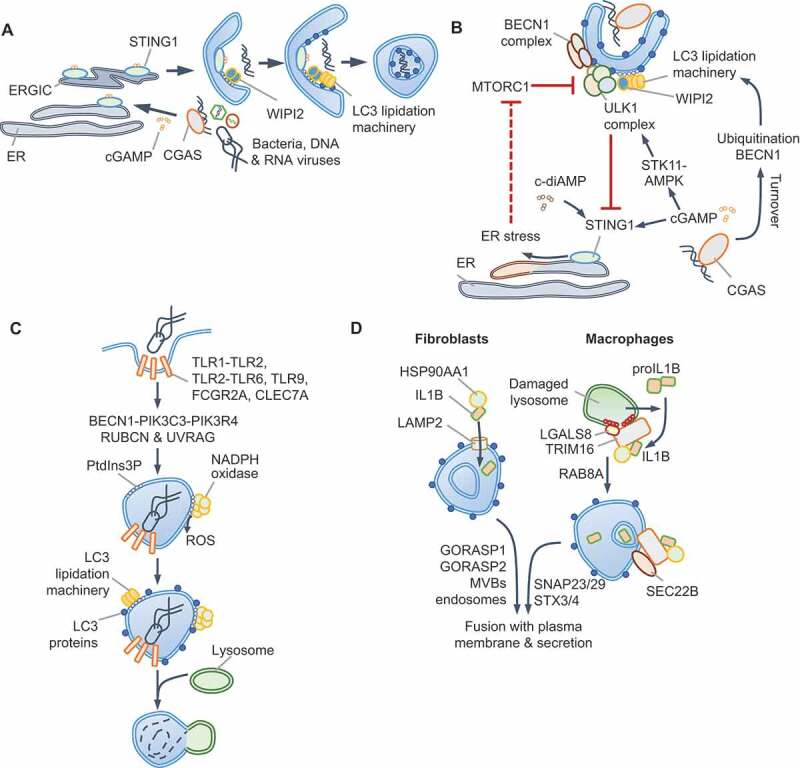
Autophagy via CGAS-STING1 and non-canonical roles of autophagy proteins. (A) ULK1-independent autophagy via STING1. The detection of dsDNA by CGAS generates cGAMP that binds and activates STING1 in the ER. Trafficking of STING1-cGAMP through the ERGIC promotes PtdIns3P production independently of ULK1 and BECN1-PtdIns3K-C3 complexes. The PtdIns3P-binding protein WIPI2 assists in ATG5-dependent LC3 lipidation and autophagosome formation. CGAS and STING1 are turned over within autophagosomes. A separate subcellular compartment containing STING1 and TBK1 (not shown) promotes type I IFN signaling. (B) ULK1-dependent STING1 and CGAS turnover. cGAMP produced by CGAS can induce STING1-dependent autophagy via CGAS ubiquitination or BECN1 interactions and through cGAMP-mediated STK11/LKB1-AMPK activation. The detection of c-diAMP, produced by Gram-positive bacteria such as *Listeria innocua* (not shown), promotes STING1-dependent ER-stress through unknown mechanisms. The activation of the unfolded protein response (UPR) results in EIF2S1 phosphorylation and the selective translation of ATF4 targets, such as DDIT4/REDD1, which inhibit MTORC1 (dotted red line). Autophagy is thus activated directly through ULK1 and BECN1-PtdIns3K-C3 complexes, or MTORC1 inhibition in these settings. (C) Some receptors, especially FCGR2A/FcγR2A, CLEC7A/Dectin 1 and some TLRs, use autophagy-related proteins during phagocytosis in a process that involves LC3 lipidation. In this pathway, the BECN1-PIK3C3-PIK3R4 complex containing RUBCN and UVRAG generates PtdIns3P. Phagosomes, which are enclosed by a single bilayer, are coated with LC3 and fuse with lysosomes. NADPH oxidase, whose subunits bind PtdIns3P and recruit it to phagosomes, produces ROS for LC3-associated phagocytosis (LAP). ULK1 complex, AMBRA1 and ATG14, and therefore nutrient signals, are dispensable in this process. (D) Unconventional secretion of mature IL1B. Expression of CASP1 and proIL1B in fibroblasts results in mature IL1B production upon starvation. IL1B is translocated into LC3-positive vesicles through HSP90AA1 and independently of ATG2B, RB1CC1 and ATG5 activities. This suggests CMA-like threading of IL1B directly into vesicles followed by secretion of soluble IL1B protein, which requires GORASP2/GRASP55, GORASP1/GRASP65 and multivesicular bodies (MVBs). IL1B resides within the inter-membrane space. In macrophages, lysosomal damage-induced IL1B is captured by TRIM16, which also interacts with LGALS8 and HSP90AA1 to transfer IL1B into vesicles. RAB8A is required for vesicle trafficking and the TRIM16-partner SEC22B SNARE mediates fusion of vesicles to plasma membrane through SNAP23, SNAP29, STX3 and STX4. An outstanding question is how vesicles containing cargo do not fuse with lysosomes

STING1 has an evolutionarily conserved role in initiating autophagy, from the sea anemone *Nematostella vectensis* to mammals, whereas mammalian STING1 also activates type I IFN production through TBK1 [95–97]. STING1 is activated by binding to 2ʹ,3ʹ-cGAMP (cGAMP), a cyclic dinucleotide (CDN) second messenger produced upon cytosolic DNA sensing by CGAS. For example, *M. tuberculosis* infection results in CGAS-STING1-dependent xenophagy [98–101] and DRAM1-driven autophagosome-lysosome fusion [102]. The TBK1-driven type I IFN stimulatory function of STING1 is genetically separable from STING1-driven autophagy initiation through an ATG5-ATG16L1-dependent, ULK1- and BECN1-independent pathway [95,96] (Figure 4A). STING1-dependent autophagy can thus proceed independently of nutrient-derived signals and without MTORC1 inactivation. It is therefore plausible that MTORC1-dependent anabolic pathways may not be turned off even though autophagic flux and LC3-lipidation are turned on. These findings further suggest that therapeutic interventions, for example, through small molecule activators of STING1, to promote selective autophagy, without dampening anabolic responses through MTORC1, may be possible.

It is also noteworthy that in contrast to the above studies, AMPK-STK11 and ULK1-BECN1-dependent canonical autophagy activation by CGAS-STING1 has also been reported. For instance, CGAS-STING1 activation results in STING1 turnover through increased TBK1-dependent SQSTM1 phosphorylation and autophagy maturation [103,104] and/or cGAMP-dependent activation of AMPK-STK11 and ULK1-driven autophagy [105] (Figure 4B). Some Gram-positive bacteria, such as *Listeria innocua* and *Staphylococcus aureus*, may activate STING1 through the related bacterial CDN, bis-(3ʹ-5ʹ)-cyclic dimeric adenosine monophosphate (c-di-AMP), which is a weaker agonist of human STING1 [106]. In this scenario, STING1-dependent triggering of the unfolded-protein response (UPR) results in translational shut-down and preferential translation of ATF4 due to the phosphorylation of EIF2S1 [106] (Figure 4B). It is plausible that increased translation of ATF4, which mediates the expression of MTORC1 inhibitors DDIT4/REDD1 and SESN2, is responsible for MTORC1 inhibition and subsequent stimulation of autophagy (Box 1); however, this was not tested in this study. Several questions need to be addressed to reconcile ULK1-dependent and -independent autophagy via STING1. How does cGAMP stimulate AMPK-STK11? Does weaker activation of STING1 by c-di-AMP activate UPR? Further work should determine whether STING1 can indeed stimulate both ULK1- and BECN1-independent and -dependent autophagy in different contexts or whether different experimental conditions (e.g., different cell lines, species-specific effects, treatment regimens such as purified DNA or synthetic cGAMP or bacterial infection) have led to disparate findings.

### Non-canonical roles of autophagy proteins

We now turn our attention to autophagy-independent roles of core autophagy proteins that are independent of MTORC1 or AMPK [107,108]. A prominent non-canonical role of autophagy proteins is the endocytic/phagocytic pathway called LC3-associated phagocytosis (LAP) in myeloid cells (Figure 4C). Signaling via several TLRs, CLEC7A/Dectin-1, FCGR (Fc gamma receptors) and TIMD4/TIM4 triggers LAP in response to the diverse ligands of these receptors, including microbial components, opsonized particles or dead cells [107,109]. LAP is characterized by LC3 incorporation on single-membraned compartments that contrasts LC3 deposition on double-bilayer-containing “canonical” autophagosomes. LAP proceeds independently of nutrient-signals, the ULK1 complex, and components of the PtdIns3K-C3 complex, such as AMBRA1 and ATG14. However, LAP requires the BECN1-interacting protein RUBCN/Rubicon (RUN domain and cysteine-rich domain containing, Beclin 1-interacting protein) to assemble a distinct PtdIns3K-C3 complex with UVRAG [108]. As during canonical autophagy, the ATG12–ATG5-ATG16L1 scaffold mediates LC3-lipidation on “LAPosomes”. Interestingly, the WD40 domain of ATG16L1 is necessary for LC3 recruitment during LAP, but dispensable for canonical autophagy and may allow its selective involvement in the two processes [31]. PtdIns3P accumulation also enables membrane binding of the NADPH oxidase subunit NCF4/p40^phox^, leading to ROS production required for LAP. RUBCN emerges as a key scaffold in the process by interacting with BECN1, PIK3C3 and CYBA/p22^phox^ (cytochrome b-245 alpha chain) [108]. LAPosomes also permit differential TLR signaling; for instance, TLR9 on LAPosomes selectively leads to type I IFN production by interacting with CHUK/IKKA (component of inhibitor of nuclear factor kappa B kinase complex) and LC3 for TRAF3 and IRF7 activation [110]. Additionally, LAP can suppress inflammation, promote immune tolerance and protect against microbial infection [111].

Most *Atg* genes are essential and whole-body knockouts are embryonic lethal in mice [112]; studies therefore use cell type-specific loss or hypomorphic alleles. Mice lacking *Atg5* in all myeloid cells (Lyz2/LysM-Cre *Atg5^fx/fx^* mice) are extremely susceptible to *M. tuberculosis* infection; however, mice lacking *Atg16l1, Atg7, Atg3, Atg14* or *Atg12* in similar cells are not [113]. Polymorphonuclear (PMN) cell-specific loss of *Atg5* revealed that neutrophil ATG5 activity is essential to prevent their pathological recruitment to lungs and limit tissue damage. Therefore, even though autophagy plays a role in restricting *M. tuberculosis* replication in isolated macrophages, ATG5 has unique autophagy-independent role(s) in neutrophils that are protective against *M. tuberculosis*. Further studies are needed to better understand this function and its broader involvement in protecting against other infections characterized by neutrophil influx.

### Unconventional protein secretion and autophagy

Autophagy-like routes of vesicle trafficking can be adopted for the secretion of proteins that lack a signal peptide, such as IL1B (interleukin 1 beta), IL18 and lysozyme. These proteins become enclosed within autophagosome-like LC3-positive vesicles that fuse with the plasma membrane for secretory autophagy. Starvation or lysosomal damage trigger CASP1-dependent processing of pro-IL1B into mature IL1B in macrophages, leading to IL1B release that partially relies on autophagy proteins [114]. Two models have been proposed for IL1B incorporation within LC3-positive vesicles. In macrophages, IL1B is captured into vesicles by TRIM16 in a process that involves LGALS8, HSP90AA1/HSP90 (heat shock protein 90 alpha family class A member 1) and the SNARE SEC22B [83,114] (Figure 4D). On the other hand, overexpression of CASP1 and pro-IL1B in fibroblasts results in IL1B release through multi-vesicular body (MVB)-like intermediates through the actions of HSP90AA1, GORASP2/GRASP55 (golgi reassembly stacking protein 2), GORASP1/GRASP65 and TSG101 (tumor susceptibility gene 101) [114,115]. Notably, although LC3 is involved, proteins essential for autophagosome formation, e.g., ATG2, RB1CC1 and ATG5, are dispensable. GORASP2 is regulated by glucose levels and O-GlcNAcylation and can thus respond to nutrient signals [116,117]. The involvement of HSP90AA1 and MVBs in IL1B release from reconstituted fibroblasts suggests a **chaperone-mediated autophagy** (**CMA**, see Glossary)-like process (Figure 4D). Mutation of sequence motifs in IL1B (_127_LRDEQ_131, 132_QKSLV_136_) related to KFERQ motifs found in CMA-substrates reduces IL1B release [115]. However, as K133 is also the site for K63-ubiquitination of IL1B, leading to its maturation and release from macrophages [118], further work is necessary to validate CMA involvement. Furthermore, the involvement of GSDMD (gasdermin D), which is essential for IL1B release from myeloid cells [119], in unconventional secretion also needs to be addressed.

Studies on the secretion of lysozyme have also suggested unconventional mechanisms. Paneth cells are specialized cells in the crypts of the small intestine that produce antimicrobial peptides and have large vacuoles containing lysozyme, which can hydrolyze bacterial peptidoglycan. These lysozyme-containing vacuoles are LC3-positive during *Salmonella* infection, reminiscent of unconventional secretion. Notably, such vacuoles are absent in *Atg16l1^T300A^* knock-in mice expressing ATG16L1 with a T300A mutation that reduces its stability and mimics the human Crohn disease-linked variant [120,121], which points to an involvement of the LC3-lipidation machinery. The lack of suitable Paneth cell lines has hindered further molecular characterization of the role of autophagy in lysozyme secretion, which requires IL22 and innate lymphoid cell 3 (ILC3) function and DC-intrinsic MYD88 signaling *in vivo*.

Why does the cargo does not fuse with lysosomes? Is LC3-enclosed IL1B released from living macrophages [122]? How does autophagy promote IL1B release on the one hand and turn-over inflammasome signaling proteins on the other? Future work on secretion of IL18, which is naturally expressed in many non-myeloid cells, may provide new insights. Taken together, autophagy proteins moonlight in unconventional secretion; however, several questions remain to be addressed in the future.

## Mutations in nutrient sensing and autophagy genes

The importance of MTOR signaling and autophagy in maintaining cellular homeostasis is underscored by disease association of mutations in genes in these pathways. The PI3K-AKT1-MTOR pathway is often linked to abnormal tissue growth, tumors and cancer [123]. Mutations in the autophagy-related genes are more frequently associated with susceptibility to infection, inflammatory disease (for example, inflammatory bowel disease) and neurodegenerative diseases (Table 1) [124]. Even though most of these genes have broad expression patterns – for example, *SQSTM1, ATG16L1* and *RPTOR* are expressed in many tissues and cell types – diseases linked to them are often tissue-specific. Our understanding of the precise mechanisms of disease progression and context-specific alteration in their function is currently incomplete. Mutations in multifunctional genes, such as *SQSTM1, RIPK1, CASP8, TBK1*, among others, may compromise the crosstalk between autophagy and MTORC1. This suggests that a better understanding of context-specific regulation of these pathways will assist in the design of selective therapeutics in the future.

## Modulation of nutrient sensing and autophagy by pathogens

**Figure 5.**
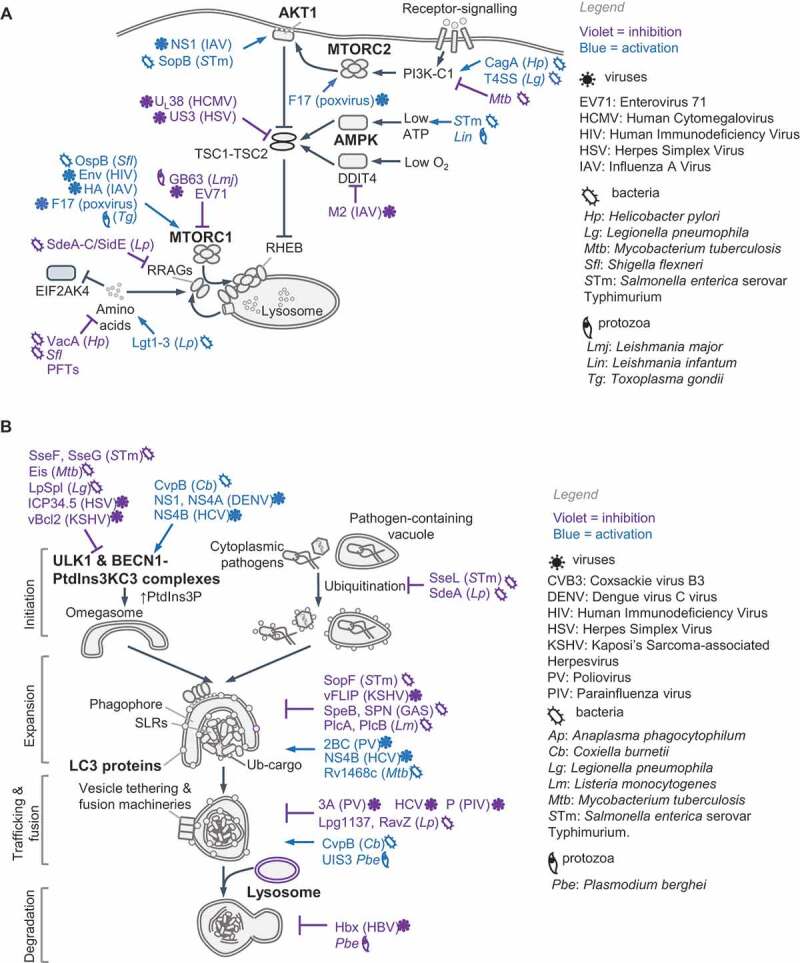
Pathogen-driven manipulation of host nutrient sensing and autophagy. Schematics in (A) and (B) show proteins used by microbial pathogens (within parenthesis) that act at the indicated steps. Pathogen-driven activation or inhibition are colored in blue and violet, respectively. See text for details and compare with **Figure 1**, which depicts core-pathways (blue arrows/lines). (**A**) Pathogens manipulate MTOR signaling through RRAGs and/or RHEB, AMPK or affect EIF2AK4/GCN2 signaling. Activation of MTORC1 promotes membrane synthesis for maintaining intracellular microbial vacuoles, biogenesis for building blocks, selective protein translation and gene transcription. Inhibition of MTORC1 blocks the host from utilizing nutrients, prevents anti-microbial gene transcription and induces autophagy. Some pathogens affect these pathways temporally and use other effectors to additionally manipulate autophagy, as shown in (B). See refs [44,125–149]. PFT: Pore Forming Toxin; SLO: streptolysin O; T3SS: Type III Secretion System; T4SS: Type IV Secretion System. (**B**) Several pathogens upregulate or inhibit xenophagy to avoid the harsh lysosomal environment. Intracellular pathogens can block the fusion of vacuoles with lysosomes or alter lysosomal contents to develop a less hostile niche and promote fusion with endosomes and/or detoxified lysosomes to obtain nutrients. See refs [150–173]

Bacteria, viruses and parasites attempt to exploit host transcription and translation controlled by MTORC1 or AMPK, and at the same time, prevent autophagy-mediated lysosomal targeting. We discuss recent work on direct manipulation of MTOR, AMPK and autophagy that illustrates how both pathways could be operating simultaneously, especially when pathogens are able to manipulate their antimicrobial effects (Figure 5A and 5B).

### Manipulation of nutrient sensing pathways

Most pathogens regulate MTORC1 temporally, either directly through a state of starvation or indirectly through PI3K-C1, AKT1 or MTORC2. The overall goal appears to shape a favorable gene expression and metabolic niche. A particularly effective inhibitor mechanism involves the proteolysis of MTOR by *Leishmania major* GB63 protein [125]. Amino acid starvation during *Shigella flexneri* inactivates MTORC1 and activates EIF2AK4, which results in an ATF3-dependent transcriptional signature e.g., increased CHAC1 and CHOP/DDIT3 [126]. Starvation can also result from pore-forming toxins that affect cellular ionic gradients and amino acid import [127–129]. *Salmonella* infection activates AMPK, which the bacterium rapidly targets for lysosomal degradation [44,130,131] to suppress autophagy initiation. In contrast, the protozoan pathogen *Leishmania infantum* activates the AMPK-STK11 axis to enhance host metabolism and its own growth [132]. Further studies should focus on mechanisms of pathogen-induced acute nutrient and energy starvation that activate EIF2AK4 and AMPK (Figure 5A).

Free amino acids accumulate after a block in protein translation resulting from the modification and inhibition of EEF1A1 by the glucosyltransferases Lgt1-Lgt3 from *Legionella pneumophila* [133]. However, concurrent ubiquitination and inhibition of RRAGB and RRAGD by SdeA-C/SidE ubiquitin ligases effectively “blinds” MTORC1 to the presence of amino acids, which we presume become available to the pathogen [133]. Late MTORC1 activation during infection promotes lipid biosynthesis to support growing microbial vacuoles, as exemplified by *L. pneumophila* and the protozoan pathogen *Toxoplasma gondii* [134–137] (Figure 5A). Therefore, temporal control of these pathways is key for intracellular pathogens.

*Shigella flexneri*-encoded effector OspB can also activate MTORC1 via the scaffolding protein IQGAP1, leading to increased cell proliferation at the site of infection. This allows replacement of dying cells and therefore provides additional intracellular niches for the infecting bacteria [138]. Indirect MTORC1 regulation is exemplified by PI3K-C1 activation by *Helicobacter pylori* [139] and *L. pneumophila* [134,135], and inhibition by *M. tuberculosis* [140], AKT1 activation by *Salmonella* [141] and influenza virus [142–144], TSC2 inhibition by HSV1 and HCMV1 [145,146], and regulation of both MTOR complexes by a poxvirus protein that acts on RPTOR and RICTOR [147,148] (Figure 5A). Typically, RNA viruses inhibit MTORC1 as they use 5ʹ-CAP-independent translation, whereas DNA viruses promote MTORC1 activation [149].

In summary, although the hijacking of most steps in these pathways is known, manipulation of nutrient sensing is context- and pathogen-specific because pathogens have unique niches and energy requirements. As discussed in the next section, pathogens employ various mechanisms to evade autophagy activated in response to their interference in nutrient sensing.

### Manipulation of autophagy

Various steps of autophagy, including initiation, capture of cargo within phagophores and maturation of autophagosomes and their fusion with lysosomes, can be targeted during infection. We refer to previous reviews on these topics [150–152] and discuss more recent studies below. *Shigella, Listeria monocytogenes* and Vaccinia virus evade capture within autophagosomes by using actin-based motility [153,154]. Antimicrobial responses against *Shigella* can be mobilized by IFN-inducible GBPs, which in turn are ubiquitination by bacterial IpaH9.8 resulting in their degradation [155–157]. Deubiquitination of microbial vacuoles prevents detection by SLRs and helps *Salmonella* and *Legionella* evade autophagy [158] (Figure 5B).

Direct inhibition of the ULK1 or BECN1-PtdIns3K-C3 complexes by parasites, bacteria and viruses suppresses autophagy initiation (Figure 5B). *T. gondii* can indirectly suppress autophagy by stimulating EGFR signaling, which activates MTORC1; however, the host can turn on autophagy upon exposure to CD40 and TNF [159] or through immunity-related GTPases (IRGs) and GBPs after IFNG stimulation [159]. Host-derived signals can thus overcome microbial virulence strategies.

ULK1-independent autophagy initiation can also be blocked, for example, by *Salmonella* SopF, a phosphoinositide binding ADP-ribosylase that modifies the V-ATPase and inhibits the recruitment of ATG16L1 and LC3-lipidation machinery [84,160]. Absence of SopF expression dampens *Salmonella* virulence *in vivo*, underscoring the important antimicrobial role of this ULK1-independent pathway. Whether the V-ATPase in involved in sensing infection by other intracellular bacteria, likely through disruption of vacuolar compartments, remains to be seen. In addition, the ULK1-independent process of LAP is inhibited by *M. tuberculosis* through CpsA, which blocks the NADPH oxidase [161] (Figure 5B).

Manipulating nutrient sensing necessitates mechanisms to avoid xenophagic lysosomal degradation, and indeed many pathogens suppress autophagy maturation. *Salmonella* modifies the potency of lysosomes by manipulating endosomal trafficking, for example, through the actions of the effector SifA, which interacts with PLEKHM1 and PLEKHM2/SKIP which, along with RUBCNL/PACER, are proteins with a RUBCN-like C-terminal domain (Rubicon homology domain) [162,163]. *L. pneumophila* uses RavZ to delipidate LC3 and the Lpg1137 protease to cleave STX17 (syntaxin 17) and avoid lysosomal fusion [164,165]. The parainfluenza virus phosphoprotein P blocks autophagosome-lysosome fusion by inhibiting the interaction between STX17 and SNAP29 [166] (Figure 5B).

Conversely, some pathogens may enhance autophagy to create a replicative niche, evade cytosolic immune detection, promote egress from cells or to exhaust antimicrobial effector mechanisms. For instance, *L. monocytogenes* listeriolysin O (LLO) promotes NLRX1-driven mitophagy, which reduces cellular ROS and enhances bacterial survival [167]. This also indicates that elevated selective autophagy of a key host organelle can benefit pathogens and that drugs that nonspecifically increase bulk autophagy may not be beneficial in such scenarios. Surprisingly, the *M. tuberculosis* surface protein Rv1468c binds ubiquitin and recruits SQSTM1, which could dampen inflammation [168]. *Coxiella burnetii* survives within large acidic LC3-positive vacuoles and uses the autophagosome-lysosome STX17 for vesicle fusion and the CvpB/Cig2 effector to manipulate PtdIns3P levels (Figure 5B) [169–171]. A similar strategy is used by *Plasmodium* through its UIS3 protein [172,173]. In summary, pathogens selectively block or activate steps within the autophagy pathway to optimize their intracellular stay.

## Summary and outlook

During starvation, autophagy helps scavenge and recycle nutrients. Nutrient sensing and starvation-induced autophagy therefore cross-regulate each other. Here, we have presented an integrated view of these processes and their concurrent use in innate immunity, where nutrients may not necessarily be limiting and may serve as second messengers. Innate immune responses deploy MTORC1 and MTORC2 to optimize transcription and translation, and autophagy for antimicrobial defense. Studies indicate that during host-pathogen interactions, the stringent feedback regulation of ULK1 and MTORC1 may be supplanted by novel regulatory hubs that permit both pathways to be turned on simultaneously. Differential activation of autophagy and MTORC1 by SQSTM1/p62^TRM^, TBK1, TRAF6 and RALB, which are among the prominent molecules involved in both pathways, needs to be studied further in disease and/or infection-relevant settings.

Work from both fields has revealed signaling mechanisms in the host whose subversion by microbial virulence factors underscores their immune roles. The PI3K-C1-MTORC2 axis is activated by PRRs and IFNs, and temporally manipulated by pathogens. Optimal MTORC1 signaling requires healthy lysosomes, for example, during signaling by endosomal TLRs. Not surprisingly, bacterial effectors that act on endosomal trafficking, lysosomes or RRAG GTPases have also been identified. The host can initiate xenophagy by targeting ULK1 to pathogen-containing vacuoles, which in turn evade outcomes of xenophagy by interfering with autophagy initiation or maturation. Importantly, ULK1-independent, and therefore nutrient and MTORC1-independent, xenophagy can be triggered by STING1 and V-ATPase by directly recruiting the LC3 lipidation machinery.

It is plausible that dual activation of MTORC1 and AMPK is more widespread, and the underlying mechanisms have not yet been identified. The diseases linked to mutations in genes in these pathways further suggest their broad yet context-specific roles in homeostasis, immunity and inflammation. Future studies should focus on deciphering the precise molecular regulatory mechanisms with a view to understanding them better and designing effective therapies for inflammatory and infectious diseases.

### Box 1

Activation of MTORC1 by amino acids and growth factors. Amino acids are sensed by proteins that can transduce the signals to the RRAGA/RRAGB-RRAGC/RRAGD complex, which is a heterodimer of GTP-bound RRAGA or RRAGB and GDP-bound RRAGC or RRAGD in its active form. RRAGs then interact with the MTORC1 subunit RPTOR/Raptor and promote its localization to lysosomes, which is essential for MTOR kinase autophosphorylation and activation. In the absence of RRAGA and RRAGB, the amino acid glutamine can also stimulate MTORC1 localization to lysosomes through ARF1 [174]. The **RAGULATOR** (see Glossary) complex, which has guanine nucleotide exchange factor activity toward RRAGA/RRAGB, and the FLCN (folliculin)-FNIP1/2 complex, which has GAP activity for RRAGC/RRAGD, positively regulate MTORC1. GAP activity toward the RRAG GTPases 1 (**GATOR1**, see Glossary) complex inhibits MTORC1 and is itself inhibited by **GATOR2** (see Glossary). The **KICSTOR** (see Glossary) complex interacts with and positively regulates GATOR1 at lysosomes and thus inhibits MTORC1. At the lysosomal surface, MTORC1 interacts with the V-ATPase and the amino acid-transporter SLC38A9. Normal lysosomal function is therefore essential for MTORC1 activity. As shown in the figure, different proteins can detect specific amino acids and stimulate MTORC1 [175,176]. Localization of MTORC1 to the lysosomes promotes its interaction with RHEB, a lysosome-localized GTPase that is activated by growth factors (e.g., insulin, EGF), TLRs and cytokine receptors. RHEB is inhibited by its GAP TSC1-TSC2 (tuberous sclerosis 1/2) [175]. Plasma membrane receptor signaling to MTORC1 is coupled via the MTORC2-AKT1-TSC1-TSC2 signaling axis (Figure 1A). Active RHEB and RRAGs are both needed for MTORC1 activation in response to growth factors. AKT1 phosphorylation also inhibits AKT1S1/PRAS40, a negative regulator that binds RPTOR [123,176]. In addition to AKT1, MAPK1/ERK2, MAPK3/ERK1 and RPS6KA1/RSK1 inhibit TSC2, thus activating MTORC1, whereas AMPK, DDIT4/REDD1 and GSK3B/GSK3β activate TSC2 [176].

**Figure:**
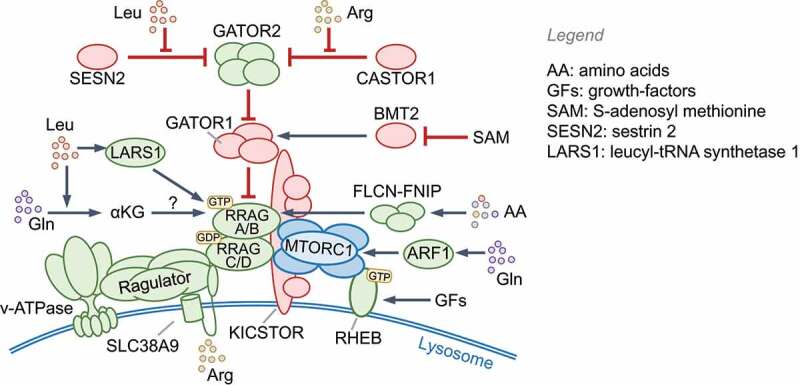

### Box 2

Signaling outcomes of MTORC1 and MTORC2 kinase complexes. Among the best-known substrates of MTORC1 are RPS6KB1 (ribosomal protein S6 kinase B1) and EIF4EBP1/4E-BP1 (eukaryotic translation initiation factor 4E binding protein 1). MTOR autophosphorylates on Ser2481, which can be found in both MTORC1 and MTORC2. In addition, RPS6KB1 phosphorylates MTOR on Ser2448, which reflects active MTORC1 and nutrient availability [177]. MTORC1 is also phosphorylated on Ser1261 in the presence of amino acids. p-RPS6KB1 stimulates protein translation through ribosomal subunit RPS6 phosphorylation and p-EIF4EBP1 fails to block EIF4E-dependent 5ʹ-CAP mRNA translation; both processes lead to increased protein translation. MTORC1 activates SREBF1 (sterol regulatory element binding transcription factor 1) and SREBF2 for lipid and cholesterol biogenesis, HIF1A (hypoxia inducible factor 1 subunit alpha) for glucose metabolism and glycolysis, ATF4 (activating transcription factor 4) and CAD (carbamoyl-phosphate synthetase 2, aspartate transcarbamylase, and dihydroorotase) for nucleotide synthesis, and PPARGC1A (PPARG coactivator 1 alpha) for mitochondrial function [123,176]. GSK3B (glycogen synthase kinase 3 beta), an inhibitor of SREBF1 and HIF1A, is inhibited by MTORC1 [177]. Phosphorylation of TFEB (transcription factor EB) retains it in the cytoplasm and prevents the expression of lysosomal and autophagy genes [26,175]. Cell proliferative actions of MTORC1 and AKT1 proceed through increased *CCND1* (cyclin D1) transcription and suppression of FOXO transcription factors, CDKN1A/p21 and CDKN1B/p27 [177]. In addition to reducing the expression of autophagy and lysosomal genes, MTORC1 inhibits autophagy by phosphorylating and inhibiting ULK1 and AMBRA1, which is a component of the BECN1-PtdIns3K-C3 complex (see Box 3 and Figure 1A) [178]. MTORC1 phosphorylates and inhibits AMPK, which also results in the suppression of autophagy because ULK1/2 and BECN1-PtdIns3K-C3 complexes are activated by AMPK. The crosstalk between AMPK and MTORC1 also includes AMPK-mediated inhibitory phosphorylation of RPTOR and the activating phosphorylation of TSC2 [26,178]. MTORC2 predominantly contains Ser2481-phospohrylated MTOR. MTORC2 phosphorylates AKT1 (Ser473, an indicator of active MTORC2), which promotes cell survival and proliferation, protein kinase C (PKC) that regulates cell migration, and the serum SGK1 (glucocorticoid-regulated kinase), which promotes cell survival and ion transport [97,123,176].

**Figure:**
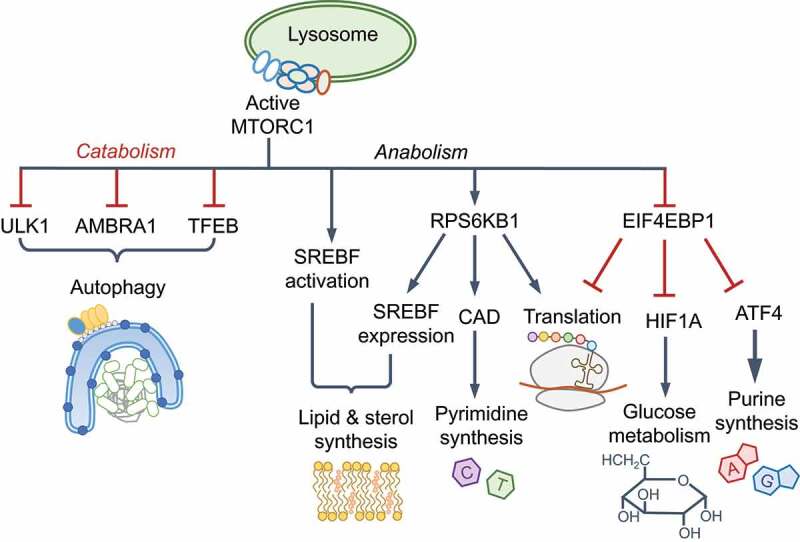

### Box 3

Starvation-induced autophagy. Starvation-induced autophagy relies on several multiprotein complexes defined by their key components (Figure 1B): (1) the ULK1/2 complex that contains ULK1 (or ULK2), RB1CC1, ATG13 and ATG101; ULK1 is activated by AMPK and inhibited by MTORC1; (2) BECN1-PIK3C3-PIK3R4 complex with AMBRA1 and ATG14; PIK3C3 is a PtdIns3K lipid kinase of catalytic class III (PtdIns3K-C3) that generates phosphoinositol-3-phosphate (PtdIns3P) and is activated by BECN1; (3) ATG9 resides in vesicles that likely mark sites for phagophore initiation and/or expansion, WIPI1/2 and ZFYVE1 PtdIns3P-binding membrane scaffolds, along with ATG2A/2B, facilitate the recruitment of the ATG16L1 complex; (4) ATG16L1 and ATG12–ATG5 (formed through the actions of ATG10-ATG7) complex promotes phagophore expansion and lipidation of the Atg8/MAP1LC3 (microtubule-associated proteins 1A/1B light chain 3)-family proteins; (5) ATG4A/B protease activity enables ATG3-ATG7 proteins to catalyze phosphatidylethanolamine crosslinking to the C-terminus of LC3-family proteins (LC3A, LC3B, LC3B2, LC3C, GABARAP and GABARAPL1/2); ATG4A/B action is reversible and can lead to LC3 delipidation; (6) selective cargo-receptors such as SQSTM1/p62-like receptors (SLRs) that can bind cargo, LC3 proteins and/or other autophagy complexes [85,179,180]. The fusion of autophagosomes to endosomes and lysosomes requires proteins that participate in endocytic trafficking, such as UVRAG (UV Radiation Resistance Associated), homotypic fusion and protein sorting complex (HOPS) and soluble NSF-attachment protein (SNAP)-receptor (SNARE) [26,27]. RAB7A-dependent activation of the HOPS complex promotes trafficking and tethering of autophagosomes and fusion via SNARE STX17 on autophagosomes and SNAP29 and VAMP8 on lysosomes [162].

## Glossary (in alphabetical order)

AMPK: AMPK is a trimeric complex of the catalytic subunit alpha and regulatory subunits beta and gamma. AMP (or ADP) can bind to the gamma subunit resulting in a conformational change that make AMPK a better substrate for phosphorylation by the constitutively active kinase STK11/LKB1.

Atg8/LC3 family proteins: a family of seven human proteins (GABARAP, GABARAPL1, GABARAPL2, MAP1LC3A, MAP1LC3B, MAP1LC3B2, and MAP1LC3C) that contain a ubiquitin-like fold and undergo covalent modification with phosphatidylethanolamine on a C-terminal glycine residue.

Chaperone-mediated autophagy: Delivery of proteins containing sequence motifs recognized by HSPA8 and co-chaperones to lysosomal LAMP2, leading to their translocation and degradation within lysosomes.

EXOCYST complex: An octameric complex that mediates the tethering of secretory vesicles prior to their fusion to plasma membrane. In humans it consists of EXOC1-8 proteins and is regulated by RALA, RHO and RAB GTPases and MAPKs.

GATOR1: A trimeric complex comprising of DEPDC5, NPRL2, and NPRL3 with GAP activity for RRAGA and RRAGB, thus a negative regulator of MTORC1.

GATOR2: A complex consisting of MIOS, WDR24, WDR59, SEH1L, and SEC13 that inhibits GATOR1.

KICSTOR: A complex containing of KPTN, ITFG2, C12ORF66, and SZT2 that tethers GATOR1 to the lysosomal surface and serves as a negative regulator of MTORC1.

Macroautophagy: is the major form of autophagy and involves the engulfment of cytoplasmic contents, organelles or pathogens within double-membraned vacuoles that fuse with lysosomes.

MTORC1: A complex containing the MTOR serine/threonine kinase and the following subunits: RPTOR/Raptor (regulatory associated protein of MTOR complex 1) and MLST8, AKT1S1/PRAS40 (AKT1 substrate 1) and DEPTOR (DEP domain containing MTOR interacting protein). RPTOR promotes substrate recognition and lysosomal localization, MLST8 promotes phosphorylation, AKT1S1 and DEPTOR negatively regulate MTORC1.

MTORC2: A complex containing MTOR and the following proteins: RICTOR (RPTOR independent companion of MTOR complex 2), DEPTOR, MLST8, PRR5/Protor-1, PRR5L/Protor-2) and MAPKAP1/SIN1 (MAPK associated protein 1). RICTOR promotes substrate recruitment and MAPKAP1, PRR5 and PRR5L are regulatory subunits.

PtdIns3K class III complex: A class III PtdIns3K complex consisting of PIK3C3/VPS34 (contains PtdIns3K activity), PIK3R4/VPS15, BECN1 and either ATG14 for autophagic roles or UVRAG when involved in endocytic trafficking. Several accessory proteins regulate these complexes (e.g., AMBRA1). These complexes generate phosphatidylinositol-3-phosphate (PtdIns3P) for membrane expansion.

RAGULATOR: a GTP-exchange factor for the RRAGA/RRAGB GTPases and consists of LAMTOR1-5. RAGULATOR tethers RRAGs to lysosomal membranes in the proximity of the lysosomal V-ATPase complex and activates MTORC1.

TSC1-TSC2: Heterotrimeric complex comprising of TSC1, TSC2, and TBC1D7 with GAP activity toward RHEB GTPase that negatively regulates MTORC1.

SLRs: sequestosome 1-like receptors, such as SQSTM1/p62, NBR1, CALCOCO2/NDP52 and TAX1BP1, are proteins that recognize ubiquitinated cargo via their ubiquitin-binding domain (UBA) and contain an LC3 interacting region (LIR) that enables targeting of cargo to autophagy.

ULK1 complex: The canonical autophagy initiation complex, comprising of ULK1, ATG13, RB1CC1 and ATG101. It nucleates the phagophore by phosphorylating and activating components of the BECN1-class III PtdIns3K (PtdIns3K-C3) complex.
